# Prevalence and associated factors of depression and anxiety among patients with melasma: a cross-sectional study in China

**DOI:** 10.3389/fpsyt.2025.1655781

**Published:** 2025-10-13

**Authors:** Wenjing Chen, Runan Fang, Kaihui Zhang, Yang Shen, Yuan Sun, Jiacheng Gao, Ye Zhai, Liuhong Sun, Jianhong Li

**Affiliations:** Dermatology Department, Dongzhimen Hospital, Beijing University of Chinese Medicine, Beijing, China

**Keywords:** melasma, depression, anxiety, prevalence, China

## Abstract

**Background:**

Melasma is a common acquired skin hyperpigmentation disorder characterized by light to dark brown macules and patches, predominantly on the face. Due to its visible nature, the condition often imposes substantial psychological and emotional burdens on affected individuals. Depression and anxiety are common conditions that occur in patients suffering from melasma; however, comprehensive data regarding their prevalence and associated factors remain scarce. This cross-sectional observational study aimed to assess the prevalence of depression and anxiety in patients with melasma and identify potential associated factors.

**Methods:**

A total of 264 melasma patients were recruited for the study between July 2023 and May 2024. Depression and anxiety were assessed using the Self-rating Depression Scale (SDS) and the Self-rating Anxiety Scale (SAS), respectively. Univariate and multivariate logistic regression analyses were conducted to determine variables associated with depression and anxiety among patients with melasma.

**Results:**

The study revealed a prevalence of 33.3% (95% CI: 27.610-39.057) for depression and 21.6% (95% CI: 16.595-26.587) for anxiety among melasma patients. Age ≥ 45 years, high BMI and poor quality of life were identified as factors independently associated with depression in patients with melasma. Poor sleep quality was found to be an independently relative factor for anxiety among patients with melasma.

**Conclusions:**

The prevalence of depression and anxiety was higher among patients with melasma. Depression was negatively correlated with the older but positively correlated with high BMI and poor quality of life. Anxiety was positively associated with poor sleep quality. On the basis of these findings, the present study suggests that routine screening for depression and anxiety should be conducted in patients with melasma to facilitate early intervention strategies. Such targeted approaches may not only enhance treatment outcomes but also significantly improve patients’ overall quality of life.

## 1 Introduction

As the largest organ of the human body, the skin acts as the primary interface between the body’s internal physiology and external environments (1). The importance of the skin is beyond doubt, as it is the most visible of all. Healthy skin not only provides biological protection but also profoundly impacts psychological well-being acting as a reflection of one’s self-image and self-worth (2). In the contemporary digital era, where visual presentation is paramount, variations in skin color significantly influence self-esteem, personality development and social relationships (3, 4). Melasma is a common acquired focal hyperpigmentation disorder characterized by light to dark brown macules and patches symmetrically over photo-exposed areas of the face, such as the cheeks, forehead, and mandible (5). This pigmentary disorder affects men and women of all ethnicities and skin types, but it is prevalent in women of reproductive age, particularly those with darker complexions (6). Women of Asian, African, and Latin American descent classified as Fitzpatrick skin types IV to VI are predominantly affected (7). Although the precise cause of melasma remains unclear, several etiologic factors have been identified, including genetic predisposition, ultraviolet radiation and hormonal changes (8). Additionally, multiple exogenous and endogenous factors may exacerbate melasma, including the application of specific cosmetics, intake of photosensitizing medications, psychological stress, thyroid disorders, and certain systemic diseases (7). Skin disorders occurring on face frequently influence patients’ appearance and self-perception, often precipitating psychological stress and related comorbidities (9). The current literature indicates the link between skin disorders and psychological distress (3). Skin disorders have a higher prevalence of psychiatric morbidity than in the general population, with approximately one-third of the patients attending skin clinics suffering from psychological disorders (2, 10). Owing to the frequent facial involvement, associated social stigma, chronic course, long-term treatment, refractory nature and frequent relapses, pigmentary disorders are likely to further increase the risk for psychological comorbidities (11).

Depression and anxiety are among the most common and debilitating mental health conditions worldwide (12). Depression is clinically characterized by a persistent low mood or feeling of sadness, fatigue or decreased energy, and feelings of guilt or low self-worth (13). Anxiety mainly manifest as excessive and persistent feelings of worry, fear, nervousness, uneasiness, and apprehension, typically accompanied by related behavioral disturbances (14). A recent meta-analysis study reported that depression in melasma patients has a global pooled prevalence of 43.4% (15). It is reported that the prevalence of anxiety among patients with melasma demonstrates considerable variability across studies, ranging from 8 to 75% (10, 16). Although the precise neurocutaneous mechanisms remain incompletely elucidated, current evidence suggests several potential pathophysiological pathways, including common embryological origin, disorders of cortisol and pro-opiomelanocortin levels, and expression of nerve growth factor (NGF) receptors and neural endopeptidases (17–19). The nervous system and epidermis are both derivatives of the same embryonic germ layer (ectoderm), which provides a physiological basis for the interconnection between the nervous system and skin physiology (17). Melasma has a bidirectional relationship with depression and stress through the psycho-neuroendocrine pathway (2). The persistent stress caused by melasma leads to depression, whereas melanin production is affected as part of the local response to stress by the same hypothalamo-pituitary axis related to depression (20). Stress and depression increase the levels of cortisol and pro-opiomelanocortin, the precursor of melanocyte-stimulating hormone and adrenocorticotropic hormone. These have melanogenic potential, stimulating increased melanin granules in melanocytes (18). In addition, the expression of NGF receptors and neural endopeptidases in melasma lesions is increased compared with that in normal adjacent skin (19).

According to various classifications, melasma has not yet been categorized as a psychocutaneous disorder. Nevertheless, overall approach to psychocutaneous disorders is useful in this disorder too (10). There is little research examining the prevalence of depression and anxiety among patients with melasma, and no studies have yet been conducted on the independent factors associated with depression and anxiety in these patients. Thus, the present study is designed to assess the prevalence of depression and anxiety among patients with melasma and identify potential associated factors.

## 2 Materials and methods

### 2.1 Study design

This cross-sectional study was conducted on patients with melasma who attended the Dermatology Clinic, Dongzhimen Hospital, Beijing University of Chinese Medicine, Beijing, China, a tertiary hospital center, from July 2023 to May 2024. The study was approved by the Ethical Committee of Dongzhimen Hospital Affiliated to Beijing University of Chinese Medicine (2023DZMEC-293-02), and written informed consent was obtained from all participants.

### 2.2 Participants

The source population was melasma patients who attended to the Dermatology outpatient clinic of Dongzhimen Hospital. Caregivers aged >18 years who understood the written Chinese language (as all survey questionnaires were Chinese versions) were included in the study. Patients with learning disabilities, head injuries and neurological diseases (which might compromise cognitive function and comprehension ability for survey questions) were excluded from the study.

### 2.3 Sample size

To determine the prevalence of depression and anxiety, previous study by Dabas G et al. (21) was referenced. The sample size was determined by calculating using a single population proportion. The sample sizes required to estimate the prevalence of depression and anxiety were determined to be 172 and 158, respectively, based on an expected proportion (P) of 0.128 for depression and 0.116 for anxiety, an alpha (α) of 0.05, and a standard normal variable (Z) of 1.96. Considering 20% for missing data and for the non-response rate, the final sample size was 207.


$$\text{n}=\frac{{\left({\text{Z}}_{\text{α}/2}\right)}^{2}\text{P}(1-\text{P})}{{\text{d}}^{2}}$$

### 2.4 Data collection

#### 2.4.1 Questionnaire

All the participants completed a self-assessment questionnaire. Trained investigators conducted the survey and explained the purpose of the study and the guidelines for filling out the questionnaires to the participants. The questionnaire consists of the following three parts:

1. The questionnaire included questions on the socio-demographic characteristics and clinical characteristics of the patients. The socio-demographic characteristics comprised gender, age, marital status, educational level, occupational status, monthly income, and so on. The clinical characteristics contained body mass index (BMI), disease course, family history, distribution of lesions, etc.
2. Sleep quality was assessed using the Pittsburgh Sleep Quality Index (PSQI) (scores range from 0-21, with higher scores indicating poorer sleep quality). Quality of life was measured utilizing the Melasma Quality of Life (MELASQOL) scale (scores range from 7-70, with a higher score suggesting worse quality of life).
3. The Self-rating Depression Scale (SDS) and Self-rating Anxiety Scale (SAS), which were are widely used, user-friendly measurement tools for self-assessment by patients, were adopted to evaluate depression and anxiety, respectively. The participants rated how often they had experienced each symptom over the past week. Both of these scales consisted of 20 items with a 4-point Likert-type scale for each item (a little of the time, some of the time, a good part of the time, most of the time). The SDS that 10 items reflected positive experiences and 10 items reflected negative experiences rated emotional, physiological, psychomotor, and psychological imbalances (22). The SAS comprised 15 questions to evaluate increasing anxiety levels and five questions to assess decreasing anxiety levels (23). The scores of the 20 items were added, and then multiplied by 1.25 to obtain the integer of the standard points. The standardized scoring algorithm was used to define depression and anxiety, with a total score range of 25-100. The cutoff score for depression was 53 on SDS, and the cutoff score for anxiety was 50 on SAS. The validity and reliability of SDS and SAS had been examined in the Chinese population (24).

#### 2.4.2 Clinical features of melasma

The clinical features of melasma were evaluated by a single trained researcher under the direct supervision of a dermatologist.

Fitzpatrick skin phototypes was based on skin tanning ability and skin reaction to prolonged sun exposure, which classified human skin color into six categories, ranging from type I (pale white) to type VI (deeply pigmented dark brown to black) (25).

Melasma had been classified into three clinical patterns according to the distribution of lesions. The centrofacial pattern involved the central face, forehead, nose, cheeks, upper lip, and chin. The involvement of the cheeks and nose was characteristic of the malar pattern. The mandibular pattern predominantly affected the mandibular region (7).

When the skin lesion was enlarged, the color became darker, erythema, redness occurred after scratching or when lesion faded after compression with glass, it was in the active stage; in contrast, it was in the stable stage (26).

Melasma was classified into the melanized type (M type) and the melanized with vascularized type (M+V type) based on vascular involvement. The M type manifested as non-fading skin lesions after compression with glass and an increase in color contrast between the lesion and the non-lesion areas under Wood’s lamp. The M+V type was characterized by partially fading skin lesions after compression with glass, with an indistinct increase in color contrast between the lesions and non-lesions under Wood’s lamp (27).

### 2.5 Statistical analysis

Descriptive statistics were presented as mean and standard deviation (SD) or median and interquartile range (IQR) for continuous variables, and frequencies and percentages for categorical variables. All variables were entered into univariate logistic regression analysis, and those with a *P*-value < 0.15 were included in multivariate logistic regression model to individually identify associated factors of depression and anxiety in patients with melasma. Odds ratios (OR) with 95% confidence intervals (CI) were estimated, and a *P* value < 0.05 was considered statistically significant. The variance inflation factor (VIF) was used to test multicollinearity, and no multicollinearity was detected. All statistical analyses were performed using SPSS version 20.0.

## 3 Results

### 3.1 Demographic characteristics

#### 3.1.1 Socio-demographic characteristics

Of 275 patients screened, a total of 264 patients met the qualified criteria and were recruited into the study. Most of them were female (n=256, 97.0%). The age range of the patients was 24–65 years, with a median age of 40 [36,44] years. The majority of participants in our sample were Han Chinese (the major ethnic group in China) 241 (91.3%), and the rest were ethnic minorities 23 (8.7%), mainly Manchu. 185 (70.1%) participants were married and the majority of participants had children (n=162, 61.4%). 232 (87.9%) of participants had a bachelor degree or higher, and 239 (90.5%) of participants were employed. Over one-third of the patients (n=98, 37.1%) were high-income earners. (**Table 1**)

**Table 1:** Socio-demographic and clinical characteristics of patients with melasma.

Variables	Levels	Number	Percent
Gender	Male	8	3.0
	Female	256	97.0
Age group (years)	<45	211	79.9
	≥45	53	20.1
Ethnic minorities	No	241	91.3
	Yes	23	8.7
Marital status	Single	79	29.9
	Married	185	70.1
Having children	No	102	38.6
	Yes	162	61.4
Educational level	High school or less	32	12.1
	Bachelor or higher	232	87.9
Occupational status	Unemployed	25	9.5
	Employed	239	90.5
High monthly income (≥ 12,000 yuan)	No	166	62.9
	Yes	98	37.1
High BMI (≥ 25 kg/m^2^)	No	239	90.5
	Yes	25	9.5
Disease course (months)	≤36	104	39.4
	>36	160	60.6
Treatment history	No	74	28.0
	Yes	190	72.0
Family history	No	176	66.7
	Yes	88	33.3
Fitzpatrick skin phototype	II	4	1.5
	III	68	25.8
	IV	190	72.0
	V	2	0.8
Distribution of lesions	Malar pattern	202	76.5
	Centrofacial pattern	60	22.7
	Mandibular pattern	2	0.8
Clinical staging	Stable stage	181	68.6
	Active stage	83	31.4
Vascular involvement	M type	164	62.1
	M+V type	100	37.9
Poor sleep quality (PSQI > 5 scores)	No	104	39.4
	Yes	160	60.6
Poor quality of life (EMLASQOL > 40 scores)	No	192	72.7
	Yes	72	27.3

#### 3.1.2 Clinical characteristics

The mean of BMI was 21.6 ± 2.6 kg/m^2^ (ranging 16.2-30.1 kg/m^2^). The median disease duration was 60 [36,99] months, and 160 (60.6%) patients in our study had a disease course of more than 3 years. The majority of the patients (n=190, 72%) were treated previously, and 88 (33.3%) patients had a family history. Among the patients, the majority of patients 190 (72%) had Fitzpatrick skin phototype IV, followed by 68 (25.8%) cases of type III, with only 4 (1.5%) and 2 (0.8%) cases of type II and type V, respectively. Most of the patients had skin lesions distributed in the malar and centrofacial patterns, with 202 (76.5%) and 60 (22.7%) cases respectively, and only 2 (0.8%) cases were located in the mandibular pattern. The majority of patients of skin lesions were in the stable phase (n=181, 68.6%), and classified as the M type (n=164, 62.1%). The median scores for the PSQI and MELASQOL were 6 [5,9] and 29 [18,42], respectively. (**Table 1**)

### 3.2 Prevalence of depression and anxiety

According to the SDS and SAS questionnaire, the median scores for the evaluation of depression and anxiety were 46 [37,56] and 40 [33,47], respectively. The prevalence of depression and anxiety among patients with melasma assessed using the SDS and SAS questionnaire was 33.3% (95% CI: 27.610-39.057) and 21.6% (95% CI: 16.595-26.587), respectively.

### 3.3 Factors associated with depression

To identify the independent factors of depression among melasma patients, univariate and multivariate regression models were used. The univariate analysis: age group, ethnic minorities, having children, high BMI, distribution of lesions, clinical staging, poor sleep quality and poor quality of life showed a significant association with depression. After multivariate analysis, three factors were identified: patients with melasma aged 45 years and above (AOR = 0.419, 95% CI: 0.191-0.918, *P* = 0.030) had a lower prevalence of depression as compared to younger patients under the age of 45. High BMI (AOR = 2.547, 95% CI: 1.023-6.343, *P* = 0.045) and poor quality of life (AOR = 2.271, 95% CI: 1.248-4.133, *P* = 0.007) were independent factors of depression among melasma patients. (**Supplementary Table S1**)

### 3.4 Factors associated with anxiety

After including all variables in univariate and multivariate regression analyses, the univariate analysis: clinical staging, vascular involvement, poor sleep quality and poor quality of life. After multivariate analysis, the independent factor associated with anxiety in patients with melasma was poor sleep quality (AOR = 4.266, 95% CI: 1.976-9.206, *P* < 0.001). (**Supplementary Table S2**)

## 4 Discussion

In this study, we investigated the prevalence and associated factors of depression and anxiety in melasma patients. Among patients with melasma, the prevalence of depression and anxiety was 33.3% and 21.6%, respectively. The prevalence of depression in this study was similar to that observed in Greece (34.7%), while the prevalence of anxiety was comparable to that in Pakistan (19.5%) (6, 28). The prevalence of depression was found to be higher than the study finding from India 12.8%, and lower than from Brazil 43.2% and Mexico 53.0% (21, 29, 30). The prevalence of anxiety was higher than the finding from India 11.6%, but lower than from Brazil 52.2% and Mexico 62.0% (21, 29, 30). The psychological impact of melasma is profoundly shaped by cultural and social contexts, such as Brazilian culture’s emphasis on beauty (31), which may explain the observed differences in prevalence across various countries. In addition, these differences may also be attributed to variations in sample size, service setup, study participants, research duration, and sampling technique.

In the present study, we found that patients in the older age group (≥45 years) had a lower risk of depression compared to younger individuals with melasma, a finding consistent with previous research (28). Due to the high visibility of the lesions, melasma patients often felt unattractive, frustrated, and socially embarrassed. In some cases, this could be considered a social stigma (6). Younger individuals, particularly women in the stages of partner selection and family planning, generally placed greater emphasis on physical appearance and social comparison, making them more susceptible to the impact of this condition (31, 32). In contrast, older people might possess greater mental resilience, greater life experience, better emotion regulation, and more positive coping mechanisms. As time goes by, following an acceptance-over-time pattern observed in other chronic diseases, patients with melasma gradually developed better emotional adaptation and acceptance, thereby alleviating the emotional burden and stigma associated with visible skin lesions. Furthermore, elderly patients might benefit from a shift in life priorities and self-perception, which could help to mitigate appearance-related concerns. So, these factors might be the reason why younger patients with melasma were more prone to depression.

High BMI was an independent risk factor associated with depression in patients with melasma, with it showing a positive relationship with depression in the multivariable analysis. This was congruent with a previous study, with the likelihood of depression appearing to increase with increasing the BMI (33). Studies looking at genetic factors suggested that there was an overlap in genetic loci associated with both depression and BMI (34). Current evidence demonstrated a bidirectional association between obesity and depression (33). On the one hand, obesity might increase the risk of depression by body image dissatisfaction, dieting, and weight-related stigma. On the other hand, depression might increase the risk of obesity, through biological effects or indirectly through mechanisms such as binge eating, poor adherence to weight management programs, negative thoughts, and low social support (34).

The impact of skin disorders on patients’ quality of life was considered to be a more predictive factor of mental health issues than the severity of the skin condition itself (35). To better assess the quality of life in patients with melasma, we utilized the MELASQOL scale. Multivariate analysis revealed that poor quality of life was a statistically significant associated factor for depression in patients with melasma, indicating that depression was positively correlated with poor sleep quality. It was noteworthy that the association between quality of life and anxiety did not reach statistical significance. This result differed slightly from the findings of a previous Tunisia survey, which indicated that quality of life was related to both (36). One possible explanation was that the impact of melasma on quality of life (such as long-term dissatisfaction with appearance and social avoidance) might be more likely to lead to a persistent, internalized emotional state (2, 35, 37); whereas anxiety might be more readily triggered by specific, acute factors, and its association with quality of life was relatively weak. Moreover, sleep quality, as a predictor of anxiety, might play an intermediary or regulatory role in the relationship between quality of life and anxiety. The association of this discrepancy warranted exploration and validation in future research.

Sleep problems were a recognized risk factor for both physical and mental conditions (38). This study used the PQSI to assess the sleep quality of patients with melasma, and the results indicated that poor sleep quality was the only statistically significant independent predictor for developing anxiety in patients with melasma. This result was similar to previous findings (39). While other sociodemographic and clinical variables did not differ significantly in the multivariate model. This could mean that, for patients with melasma, the psychological stress induced by the illness itself triggers anxiety more through disrupting the core aspect of sleep, rather than direct action. Sleep quality and anxiety had a bidirectional relationship, mutually reinforcing and coexisting (40). Good-quality sleep played a vital role in stabilizing and regulating emotions, while long-term sleep disturbance could cause anxiety (41). The mechanism may be related to dysfunction of the 5-hydroxytryptamine (5-HT) system. 5-HT is a key neurotransmitter that regulates sleep, mood and cognition (42). Insomnia patients accompanied by anxiety typically exhibited low 5-HT levels and reduced functionality, which resulted in a decrease in excitability of neural activity in the body and induced anxiety (43). Moreover, sleep disruption and anxiety may share similar underlying neural circuit mechanisms, which provides further explanation for their close physiological connection (44). Excessive worry and rumination caused by skin lesions may be particularly pronounced at night, disrupting sleep. Poor sleep, in turn, leads to a decline in emotional control the following day and an increased susceptibility to anxiety. Anxiety and sleep problems are often intertwined, collectively impacting daytime functioning and social interactions, thereby further exacerbating psychological burdens. Future longitudinal studies are necessary to delve deeper into the causal pathways between melasma, sleep quality, and anxiety.

There were several limitations in the present study. First, it is crucial to emphasize that the cross-sectional design employed in this study cannot establish causal relationships between the relevant factors and depression/anxiety. Second, the current study was based on self-reported questionnaires to assess depression and anxiety, which, despite being well-validated in the Chinese population, are subject to recall bias and cannot replace a structured psychiatric interview for definitive diagnosis. Further research aimed at defining clear cutoff values in patients is still needed. Third, participant recruitment from a single tertiary medical center located in a high-income city in China may introduce selection bias. The sample was characterized by a high proportion of female and highly educated individuals, which may limit the generalizability of the findings to other populations. Patients seeking specialized care at a large tertiary hospital were likely to represent more severe or treatment-resistant cases of melasma. They may experience a greater disease-related psychological burden. Finally, the small sample size might affect the generalizability and representativeness of the findings that should be interpreted with caution, and future studies with larger samples are needed to confirm these results.

## 5 Conclusion

The prevalence of depression and anxiety among patients with melasma was 33.3% and 21.6%, respectively. Age, BMI and quality of life were independent factors of depression and sleep quality was the independent factor associated with anxiety in patients with melasma. Therefore, we recommend jointed screening for depression and anxiety in all patients with melasma using the SDS and SAS questionnaire, especially for those exhibiting the younger, high BMI, poor sleep quality and poor quality of life. These screening can help identify people who might require additional support and interventions for their mental health.

## Data Availability

The original contributions presented in the study are included in the article/**Supplementary Material**. Further inquiries can be directed to the corresponding author.
