# Supplementary material for: Prevalence and associated factors of depression and anxiety among patients with melasma: a cross-sectional study in China
Source: Front Psychiatry. 2025 Oct 13;16:1655781. doi: 10.3389/fpsyt.2025.1655781 (PMC12554644; doi:10.3389/fpsyt.2025.1655781)
Supplement: Supplementary file 1 [file Table1.docx]

Supplementary Material

# Supplementary Tables

Table S1 Association between variables and depression in patients with melasma

| Variables | Levels | With depression | Without depression | OR(95%CI) | *P* | AOR(95%CI) | *P* |
| --- | --- | --- | --- | --- | --- | --- | --- |
| Gender | male | 4 | 4 | Ref. | 0.319 |  |  |
|  | female | 84 | 172 | 0.488(0.119-2.001) |  |  |  |
| Age group (years) | ˂45 | 78 | 133 | Ref. | 0.015 | Ref. | 0.030 |
|  | ≥45 | 10 | 43 | 0.397(0.189-0.833) |  | 0.419(0.191-0.918) |  |
| ethnic minorities | No | 77 | 164 | Ref. | 0.128 | Ref. | 0.239 |
|  | Yes | 11 | 12 | 1.952(0.825-4.622) |  | 1.751(0.69-4.443) |  |
| Marital status | Single | 31 | 48 | Ref. | 0.184 |  |  |
|  | Married | 57 | 128 | 0.690(0.398-1.194) |  |  |  |
| Having children | No | 41 | 61 | Ref. | 0.061 | Ref. | 0.160 |
|  | Yes | 47 | 115 | 0.608(0.361-1.024) |  | 0.669(0.382-1.173) |  |
| Educational level | High school or less | 13 | 19 | Ref. | 0.352 |  |  |
|  | Bachelor or higher | 75 | 157 | 0.698(0.327-1.489) |  |  |  |
| Occupational status | Unemployed | 8 | 17 | Ref. | 0.882 |  |  |
|  | Employed | 80 | 159 | 1.069(0.442-2.583) |  |  |  |
| High monthly income (yuan) | No | 56 | 110 | Ref. | 0.857 |  |  |
|  | Yes | 32 | 66 | 0.952(0.560-1.619) |  |  |  |
| High BMI (kg/m^2^) | No | 75 | 164 | Ref. | 0.042 | Ref. | 0.045 |
|  | Yes | 13 | 12 | 2.369(1.032-5.437) |  | 2.547(1.023-6.343) |  |
| Disease course (months) | ≤36 | 33 | 71 | Ref. | 0.656 |  |  |
|  | >36 | 55 | 105 | 1.127(0.666-1.908) |  |  |  |
| Treatment history | No | 22 | 52 | Ref. | 0.439 |  |  |
|  | Yes | 66 | 124 | 1.258(0.704-2.249) |  |  |  |
| Family history | No | 61 | 115 | Ref. | 0.518 |  |  |
|  | Yes | 27 | 61 | 0.834(0.482-1.445) |  |  |  |
| Fitzpatrick skin phototype | Ⅱ | 1 | 3 | Ref. | 0.808 |  |  |
|  | Ⅲ | 26 | 42 | 1.857(0.183-18.811) | 0.600 |  |  |
|  | Ⅳ | 61 | 129 | 1.419(0.145-13.919) | 0.764 |  |  |
|  | Ⅴ | 0 | 2 | 0(0-.) | 0.999 |  |  |
| Distribution of lesions | Malar pattern | 61 | 141 | Ref. | 0.150 | Ref. | 0.170 |
|  | Centrofacial pattern | 26 | 34 | 1.768(0.977-3.196) | 0.059 | 1.73(0.901-3.323) | 0.100 |
|  | Mandibular pattern | 1 | 1 | 2.311(0.142-37.559) | 0.556 | 4.179(0.244-71.683) | 0.324 |
| Clinical staging | Stable stage | 54 | 127 | Ref. | 0.076 | Ref. | 0.238 |
|  | Active stage | 34 | 49 | 1.632(0.95-2.803) |  | 1.423(0.792-2.555) |  |
| Vascular involvement | M type | 52 | 112 | Ref. | 0.473 |  |  |
|  | M+V type | 36 | 64 | 1.212(0.717-2.047) |  |  |  |
| Poor sleep quality | No | 26 | 78 | Ref. | 0.021 | Ref. | 0.068 |
|  | Yes | 62 | 98 | 1.898(1.099-3.277) |  | 1.717(0.961-3.068) |  |
| Poor quality of life | No | 51 | 141 | Ref. | <0.001 | Ref. | 0.007 |
|  | Yes | 37 | 35 | 2.923(1.666-5.128) |  | 2.271(1.248-4.133) |  |

Table S2 Association between variables and anxiety in patients with melasma

| Variables | Levels | With anxiety | Without anxiety | OR(95%CI) | *P* | AOR(95%CI) | *P* |
| --- | --- | --- | --- | --- | --- | --- | --- |
| Gender | Male | 0 | 8 | Ref. | 0.999 |  |  |
|  | Female | 57 | 199 | 462724385.191(0-.) |  |  |  |
| Age group (years) | ˂45 | 49 | 162 | Ref. | 0.202 |  |  |
|  | ≥45 | 8 | 45 | 0.588(0.26-1.331) |  |  |  |
| ethnic minorities | No | 50 | 191 | Ref. | 0.285 |  |  |
|  | Yes | 7 | 16 | 1.671(0.652-4.283) |  |  |  |
| Marital status | Single | 15 | 64 | Ref. | 0.502 |  |  |
|  | Married | 42 | 143 | 1.253(0.648-2.422) |  |  |  |
| Having children | No | 24 | 78 | Ref. | 0.544 |  |  |
|  | Yes | 33 | 129 | 0.831(0.458-1.509) |  |  |  |
| Educational level | High school or less | 8 | 24 | Ref. | 0.618 |  |  |
|  | Bachelor or higher | 49 | 183 | 0.803(0.34-1.898) |  |  |  |
| Occupational status | Unemployed | 5 | 20 | Ref. | 0.839 |  |  |
|  | Employed | 52 | 187 | 1.112(0.398-3.106) |  |  |  |
| High monthly income (yuan) | No | 39 | 127 | Ref. | 0.329 |  |  |
|  | Yes | 18 | 80 | 0.733(0.392-1.368) |  |  |  |
| High BMI (kg/m2) | No | 52 | 187 | Ref. | 0.839 |  |  |
|  | Yes | 5 | 20 | 0.899(0.322-2.511) |  |  |  |
| Disease course (months) | ≤36 | 20 | 84 | Ref. | 0.453 |  |  |
|  | >36 | 37 | 123 | 1.263(0.686-2.327) |  |  |  |
| Treatment history | No | 16 | 58 | Ref. | 0.994 |  |  |
|  | Yes | 41 | 149 | 0.997(0.519-1.916) |  |  |  |
| Family history | No | 36 | 140 | Ref. | 0.526 |  |  |
|  | Yes | 21 | 67 | 1.219(0.661-2.248) |  |  |  |
| Fitzpatrick skin phototype | Ⅱ | 1 | 3 | Ref. | 0.999 |  |  |
|  | Ⅲ | 15 | 53 | 0.849(0.082-8.767) | 0.891 |  |  |
|  | Ⅳ | 41 | 149 | 0.826(0.084-8.147) | 0.870 |  |  |
|  | Ⅴ | 0 | 2 | 0(0-.) | 0.999 |  |  |
| Distribution of lesions | Malar pattern | 45 | 157 | Ref. | 0.932 |  |  |
|  | Centrofacial pattern | 12 | 48 | 0.872(0.427-1.781) | 0.708 |  |  |
|  | Mandibular pattern | 0 | 2 | 0(0-.) | 0.999 |  |  |
| Clinical staging | Stable stage | 34 | 147 | Ref. | 0.104 | Ref. | 0.298 |
|  | Active stage | 23 | 60 | 1.657(0.902-3.045) |  | 1.412(0.737-2.705) |  |
| Vascular involvement | M type | 30 | 134 | Ref. | 0.097 | Ref. | 0.253 |
|  | M+V type | 27 | 73 | 1.652(0.913-2.989) |  | 1.442(0.77-2.702) |  |
| Poor sleep quality | No | 9 | 95 | Ref. | <0.001 | Ref. | <0.001 |
|  | Yes | 48 | 112 | 4.524(2.11-9.699) |  | 4.266(1.976-9.206) |  |
| Poor quality of life | No | 35 | 157 | Ref. | 0.032 | Ref. | 0.109 |
|  | Yes | 22 | 50 | 1.974(1.061-3.673) |  | 1.702(0.887-3.266) |  |
